# Preparation and Corrosion Resistance Study of Electrodeposited Ni-TiN Coatings Obtained at Different Magnetic Intensities

**DOI:** 10.3390/ma19010032

**Published:** 2025-12-21

**Authors:** Chaoyu Li, Limei Luo, Hao Ma, Fei Qi, Mengyu Cao, Xue Guo, Lei Qiang, Hao Gao

**Affiliations:** 1 School of Mechanical and Electrical Engineering, Sanming University, Sanming 365004, China20230663205@fsmu.edu.cn (L.L.); 20110824@fjsmu.edu.cn (H.M.); 20230337@fjsmu.edu.cn (F.Q.); 2 School of Mechanical Science and Engineering, Northeast Petroleum University, Daqing 163318, China; cmy_gx@nepu.edu.cn (M.C.); gx_cmy@nepu.edu.cn (X.G.)

**Keywords:** Ni-TiN coatings, electrodeposition, magnetic intensity, microstructure, corrosion resistance

## Abstract

In this article, the Ni-TiN coatings deposited on the surface of Q235 steel substrate via a magnetic-assisted electrodeposition approach. The surface morphology, Ti content, phase structure, and corrosion resistance of Ni-TiN coatings were investigated using a scanning electron microscope (SEM), a transmission electron microscopy (TEM), an energy disperse spectroscopy (EDS), an X-ray diffraction (XRD) instrument, and electrochemical workstation facility, respectively. SEM images showed that the surface morphology and thickness value of Ni-TiN coatings prepared at 0.7 T were superior to those obtained at 0.3 T and 1.1 T. EDS and adhesion strength results presented that the Ti content and adhesion strength of Ni-TiN coatings was lower than those produced at 0.7 T and 1.1 T. Meanwhile, Ni-TiN coatings prepared at 0.7 T possessed the highest hardness of 817.3 Hv. XRD patterns exhibited the nickel diffraction peaks of the Ni-TiN coatings fabricated at 0.7 T were broad and low, demonstrating that the size of nickel grain was fine. In comparison to other two Ni-TiN coatings, the one manufactured at 0.7 T possessed a high corrosion potential and a low corrosion current density, illustrating its outstanding corrosion resistance. Corrosion surface morphology revealed that the obvious corrosion pits emerged on the surface of Ni-TiN coatings deposited at 0.3 T, while the obvious corrosion pits were not appeared on the surface of Ni-TiN coatings manufactured at 0.7 T. In addition, the average corrosive weight loss of Ni-TiN coatings prepared at 0.7 T possessed the lowest of 7.2 mg, indicating the excellent corrosion resistance.

## 1. Introduction

Nickel matrix coatings because of excellent physical, chemical stability and high hardness have been used to conduct surface modification and attracted domestic and foreign scholars [1,2,3]. The nickel matrix coatings are popularly applied in the various mechanical engineering applications included bearings, cylinder liners, piston rings, and gears, which are used to enhance the abrasion and corrosion resistance of mechanical components [4,5,6]. The different methods are utilized to prepare nickel matrix coatings, such as electrodeposition [7], flame spraying [8], laser cladding [9], plasma treatment [10], and electroless plating [11]. Among these fabrication approaches, electrodeposition has obvious advantages of efficiency, cost, and low residual stress, which is regarded as an ideal technique to enhance the surface performances of metal facility [12,13,14]. For example, Ren et al. [15] prepared Ni-CeO_2_ coatings at different current densities. They concluded that Ni-CeO_2_ coatings produced at 20 A/dm^2^ possessed the most dense, smooth structure and the highest hardness of 665.78 Hv. Liu et al. [16] fabricated Ni-SiO_2_ coatings at different concentrations at a nanometer and micrometer scale. They reported that Ni-SiO_2_ coatings obtained at 8 g/L of 20 nm and 2 g/L 1 μm SiO_2_ possessed the best corrosion resistance. Guo et al. [17] manufactured Ni-TiN coatings at various electrodeposition parameters. They found that Ni-TiN coatings produced at 20% duty cycle possessed a hardness of 984.1 Hv and an average worn loss of 8.78 mg/mm^2^. These findings indicated that the nickel matrix coatings prepared utilizing the electrodeposition approach was excellent.

TiN nanoparticles have been widely applied in the various fields of aeronautic, marine, agricultural, and chemical industries. Wang et al. [18] prepared pure Ni and Ni-TiN coatings via direct current electrodeposition. They found that the hardness, corrosion resistance, and thermal stability of Ni-TiN coatings were better than that of pure Ni coatings. Li et al. [19] prepared Ni-TiN coatings via pulse electrodeposition approach. They explored the influence of plating parameters on the abrasion resistance of Ni-TiN coatings and predicted them utilizing artificial neural networks. Hence, the TiN nanoparticle acted as reinforced phase to improve the performances of nickel coatings in this paper. However, the Ni-TiN coatings prepared with traditional electrodeposition usually exhibited obvious agglomeration of nanoparticles and pores on the surface. As a result, the magnetic field was introduced into electrodeposition to enhance performances of nickel matrix coatings.

Electrodeposition under an imposed magnetic field, which introduces a magnetic field to improve the deposition effect of charged particles in the electrolyte, is one of emerging electrodeposition approaches to producing nickel matrix coatings. For instance, Azizi-Nour et al. [20] investigated the existence of the magnetic field and its impact on the surface morphology and corrosion resistance of Ni-Al_2_O_3_ coatings. They concluded that the existence of the magnetic field caused the surface morphology of Ni-Al_2_O_3_ coatings to vary, from pyramidal shape to spherical shape, and corrosion resistance of coatings increased significantly. Jiang et al. [21] explored the effect of SiC concentration on the Ni-SiC manufactured at electrodeposition under an imposed magnetic field. They proposed that the abrasion resistance and corrosion resistance of Ni-SiC coatings produced at 15 g/L with magnetic field was the best. So far, scholars paid more attention to conducting the electrodeposition under an imposed magnetic field for the fabrication of nickel matrix coatings. In this article, the surface morphology, element content, phase structure, and corrosion resistance of Ni-TiN coatings was investigated using scanning electron microscope (SEM), energy disperse spectroscopy (EDS), an X-ray diffraction (XRD) instrument, and a electrochemical workstation facility, respectively. These conclusions could provide relevant experiment data and further expand the Ni-TiN coatings used in the application fields of petroleum and chemical industry.

## 2. Materials and Methods

### 2.1. Preparation

**Figure 1:**
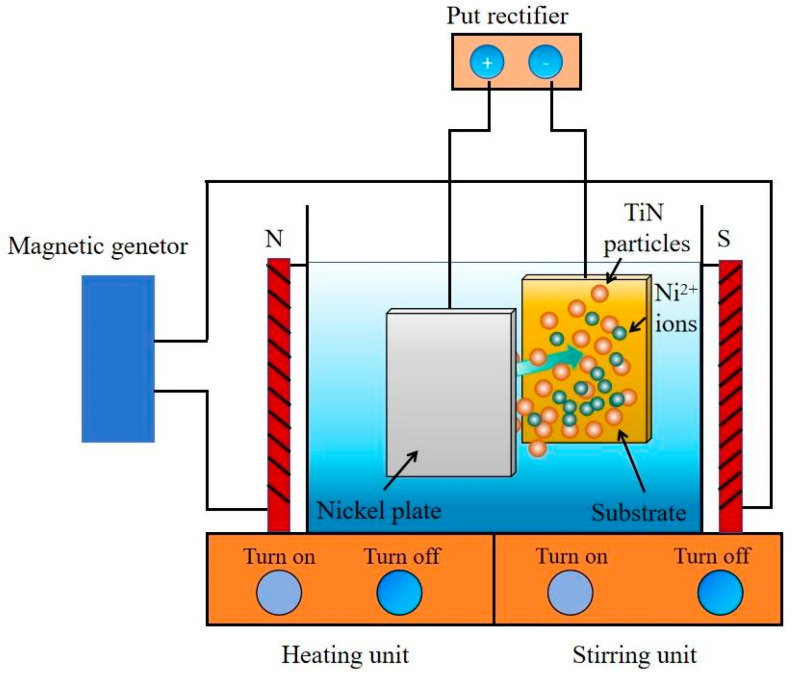
Schematic graph of manufacturing magnetic electrodeposited Ni-TiN coatings.

In this paper, the Q235 steel at a size of 30 mm × 25 mm × 3 mm was employed as a cathode and the nickel plate (purity ≥ 99.9 wt.%) with dimension of 40 mm × 30 mm × 5 mm acted as an anode. The element components of Q235 steel substrate were 0.17 wt.% C, 0.54 wt.% Mn, 0.22 wt.% Si, 0.02 wt.% P, 0.02 wt.% S, and Balanced Fe, respectively. The substrate was polished with 600, 800, and 1200 git sandpapers, respectively. Then, the substrate was washed with oil remover and distilled water. The anode and cathode were parallelly placed in the electrolyte and kept at a distance of 30 mm. Figure 1 shows the schematic graph for producing magnetic electrodeposited Ni-TiN coatings. This prefabrication system is composed of a put rectifier, magnetic field producer, stirrer, electrolyte, and heating unit. A STP-10A type put rectifier and a EMS65008A style magnetic field producer provided pulse current, voltage, and magnetic intensity for electrodeposition, respectively. Meanwhile, the magnetic field was perpendicular to the electric field and distributed uniformly. The mechanical stirrer at a speed of 120 rad/min was used to enhance the uniform distribution of TiN nanoparticles in the electrolyte, and the heating unit was employed to maintain the temperature of the electrolyte. The TiN nanoparticles were purchased from Yutai Nanotechnology Co., Ltd.(Daqing, China), at a size of approximately 20 nm. Before electrodeposition under imposed magnetic field, the electrolyte was treated by ultrasonic oscillator to reduce agglomeration of TiN nanoparticles. The pH value of electrolyte was adjusted via hydrochloric acid solution and sodium hydroxide solution. The specific electrolyte component and operation parameter used to manufacture electrodeposited Ni-TiN coatings are displayed in Table 1.

### 2.2. Characterization

The surface morphology and corrosion surface morphology of Ni-TiN coatings were investigated using scanning electron microscopy (SEM, S3400, Hitachi, Tokyo, Japan) equipped with energy-dispersive X-ray detector spectrum. The phase structure of Ni-TiN coatings was observed utilizing an X-ray diffractometer (XRD, D-Max/2500, Rigaku, Tokyo, Japan) under the conditions of Cu Kɑ, 30 kV, 0.02°/s scanning rate, and 20°–90° scanning scope. According to a previous report [22], the size (D) of nickel grain in the Ni-TiN coatings was estimated utilizing Equation (1):

(1) $$D=\frac{0.89\lambda }{\beta cos\theta }$$

where λ represents the wavelength of the X-ray, β denotes the full width at half maximum (FWHM) of the diffraction peak, and θ is the angle of Bragg.

Furthermore, the deposition rate (*S*) of Ni-TiN coatings could be calculated using Equation (2):

(2) S=T/t

where *T* and *t* were the thickness value of Ni-TiN coatings and deposition time, respectively.

The adhesion strength of Ni-TiN coatings was tested utilizing a TC-A10 type adhesion strength measurement facility under the following conditions: static pressure (30 s), applied force (100 N) and scratch length (5 mm). Moreover, the hardnesses of Ni-TiN coatings were measured using an HXD-1000 style hardness tester (Shunyu Hengping scientific instrument Co., Ltd., Shanghai, China) from five random positions and averaged.

**Figure 2:**
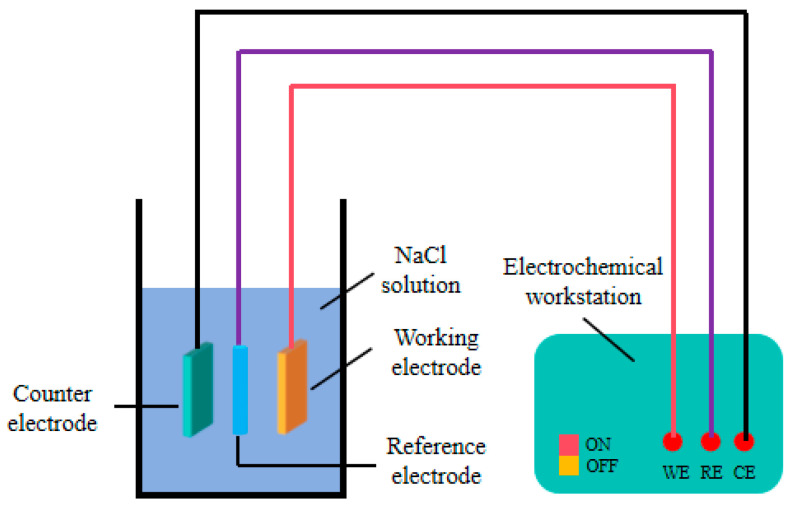
Schematic picture of three-electrode electrochemical workstation for measuring corrosion resistance of Ni-TiN coatings.

The CS350 type three-electrode electrochemical workstation was used to measure the corrosion resistance of Ni-TiN coatings immersed in 3.5 wt.% NaCl solution. Figure 2 presents the schematic picture of a three-electrode electrochemical workstation for measuring corrosion resistance of Ni-TiN coatings at room temperature. In a standard three-electrode system, the saturated calomel electrode, platinum sheet and Ni-TiN coatings were reference electrode (RE), counter electrode (CE), and working electrode (WE), respectively. Furthermore, the scanning rate was 1 mV/S, and the scanning potential range was from −1.0 V to 0.5 V. During the corrosion weight loss assessment, the Ni-TiN coatings were placed in the 3.5 wt.% NaCl corrosion liquid. The corrosive weight loss (M) of Ni-TiN coatings were calculated by Equation (3):

(3) $$M=M_{b}-M_{a}$$

where M_b_ and M_a_ represent the weight of Ni-TiN coatings before and after the corrosion test.

## 3. Results and Discussion

### 3.1. Characterization of TiN Nanoparticles

**Figure 3:**
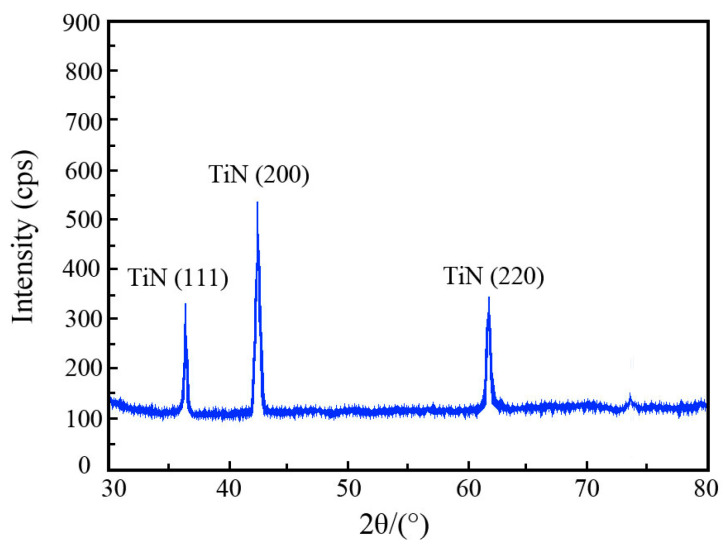
XRD pattern of TiN nanoparticles.

**Figure 4:**
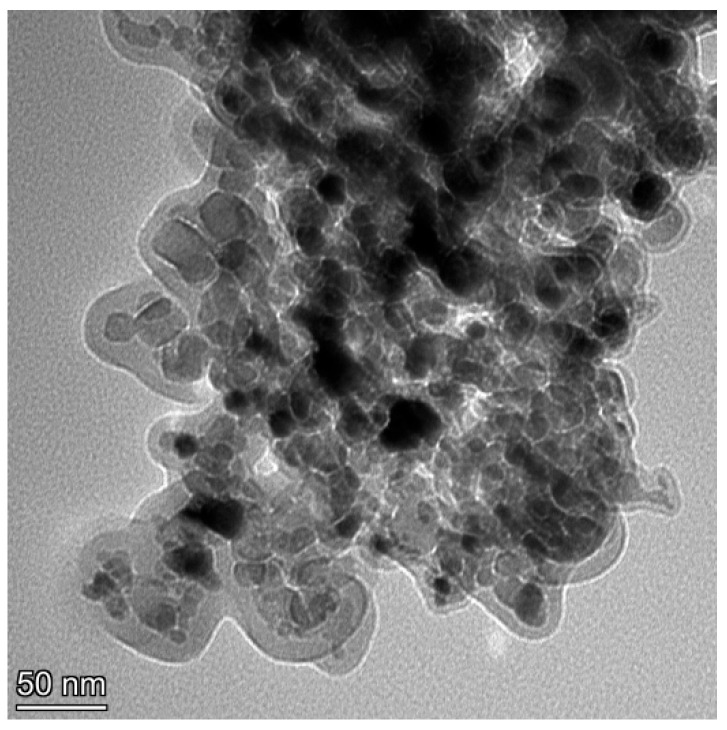
TEM image of TiN nanoparticles.

Figure 3 and Figure 4 depict the XRD pattern and TEM micro-graph of TiN nanoparticles. In Figure 3, the marked diffraction peaks of TiN nanoparticles emerged at 36.4°, 42.7°, and 61.8° corresponded to crystal planes of (111), (200), and (220), respectively [23]. From the observed result in Figure 4, it could be concluded that the shape of TiN nanoparticle was regular and partial agglomeration existed. Furthermore, the average size of TiN nanoparticles was approximately 21.8 nm, which was consistent with the dimension offered from the provider.

### 3.2. Surface Morphology and TiN Content Observation

**Figure 5:**
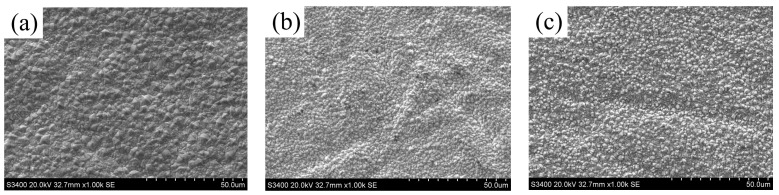
SEM images of Ni-TiN coatings produced at various magnetic intensities: (**a**) 0.3 T, (**b**) 0.7 T, and (**c**) 1.1 T.

Figure 5 depicts the surface morphologies of Ni-TiN coatings fabricated at different magnetic intensities. The observed SEM images revealed that numerous and prominent granular deposits appeared on the surface of Ni-TiN coatings produced at 0.3 T. By comparison, the surface morphology of Ni-TiN coatings manufactured at 0.7 T was dense and smooth. However, after the magnetic intensity increased to 1.1 T, the prominent granular deposits appeared on the Ni-TiN coatings again, which was smaller than the one obtained at 0.3 T.

During electrodeposition, the TiN nanoparticles with Ni^2+^ ions moved under the mutual function of magnetic field and electric field, leading to new nucleation points being formed on the surface of the cathode. However, the low or high magnetic intensity caused the agglomeration of TiN nanoparticles, which resulted in grain refinement strengthening being reduced and the generation of cell-like prominent granular deposits [24]. These coarse and irregular cell-like prominent granular deposits caused the compactness and smoothness of Ni-TiN coatings to exhibit obvious signs of decrease. By contrast, an appropriate magnetic intensity contributed to decreased agglomeration of TiN nanoparticles and enhanced grain refinement strengthening, which led to the morphology of composites becoming dense and smooth with fine-sized grains. Moreover, the appropriate magnetic intensity generated a magneto-hydrodynamic effect that contributed to the effusion of hydrogen bubble adsorbed on the surface of the cathode, which further refined grains size [25].

**Figure 6:**
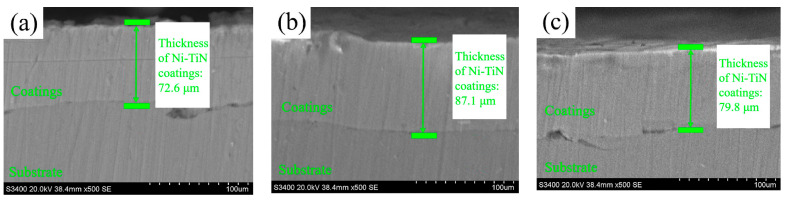
Cross-sectional thickness of Ni-TiN coatings prepared at different magnetic intensities: (**a**) 0.3 T, (**b**) 0.7 T, and (**c**) 1.1 T.

Figure 6 shows the cross-sectional thickness of Ni-TiN coatings fabricated at various magnetic intensities. It could be seen that the cross-section thickness of Ni-TiN coatings increased initially and then decreased. The cross-sectional thicknesses of Ni-TiN coatings manufactured at 0.3 T, 0.7 T, and 1.1 T were 72.6 μm, 87.1 μm, and 79.8 μm, respectively. According to the calculation of Equation (2), the deposition rate of Ni-TiN coatings were 1.82 μm/min, 2.18 μm/min, and 1.99 μm/min, respectively.

**Figure 7:**
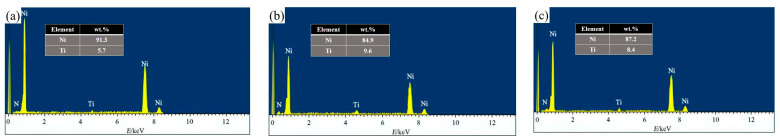
Ti content of Ni-TiN coatings prepared at different magnetic intensities: (**a**) 0.3 T, (**b**) 0.7 T, and (**c**) 1.1 T.

Figure 7 shows the Ti content of Ni-TiN coatings fabricated at various magnetic intensities. The low magnetic intensity under electric field produced weak mechanical agitation, leading to the descent probability of Ni^2+^ ions absorbed on the surface of TiN nanoparticles in the electrolyte [26]. This result caused the Ni-TiN coatings manufactured at 0.3 T to have the lowest Ti content at 5.7 wt.%. By contrast, the appropriate magnetic intensity produced fine mechanical agitation and contributed to the co-deposition of Ni^2+^ ions and TiN nanoparticles, resulting in the highest Ti content at 9.6 wt.% in the Ni-TiN coatings prepared at 0.7 T. However, when the magnetic intensity increased to 1.1 T, the mechanical agitation increased and accelerated deposition of Ni^2+^ ions. The TiN nanoparticles around the cathode surface could not be quickly replenished, leading to the Ti content of Ni-TiN coatings fabricated at 1.1 T to decrease to 8.4 wt.%.

### 3.3. Adhesion Strength Examination

**Figure 8:**
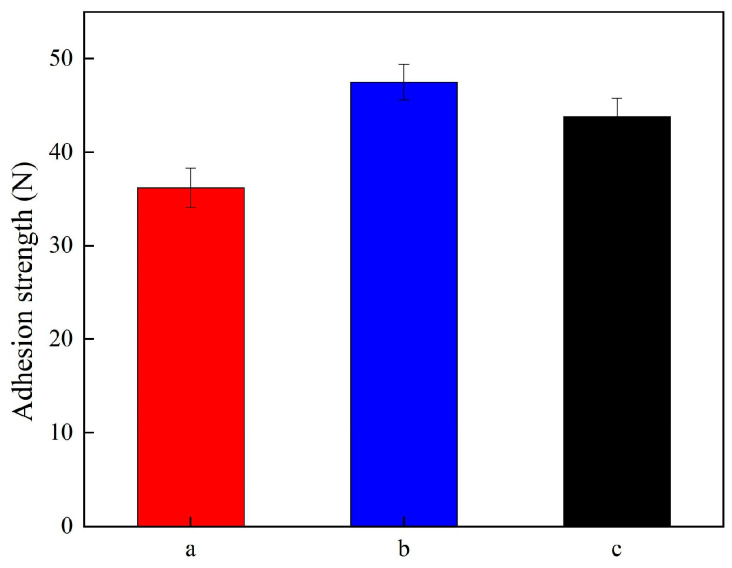
Adhesion strength of Ni-TiN coatings prepared at different magnetic intensities: (**a**) 0.3 T, (**b**) 0.7 T, and (**c**) 1.1 T.

Figure 8 shows the adhesion strength of Ni-TiN coatings produced at various magnetic intensities. The adhesion strength of Ni-TiN coatings manufactured at 0.3 T, 0.7 T, and 1.1 T were 36.2 N, 47.5 N, and 43.8 N, respectively. The reason for this result contributed to mechanical agitation and magneto-hydrodynamic effect of magnetic intensity. The appropriate magnetic intensity generated suitable mechanical agitation and magneto-hydrodynamic effect, leading to the uniform distribution of TiN nanoparticles and refined grain sizes [27]. This is conducive to the increase in the adhesion strength of coatings. In addition, the appropriate magnetic intensity could obtain a dense structure, which was beneficial in decreasing the internal stress and increasing adhesion strength of composites [28]. By contrast, the low or high magnetic intensity caused the TiN nanoparticles to agglomerate and grain refinement strengthening to decreased, causing the internal stress of coatings to increase and adhesion strength to decrease.

### 3.4. Hardness Investigation

**Figure 9:**
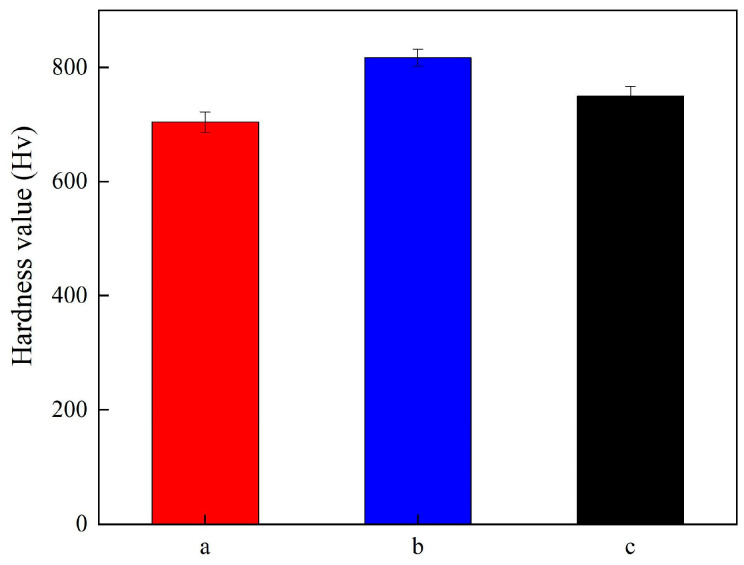
Hardness value of Ni-TiN coatings prepared at different magnetic intensities: (**a**) 0.3 T, (**b**) 0.7 T, and (**c**) 1.1 T.

Figure 9 reveals the hardness value of Ni-TiN coatings prepared at different magnetic intensities. The hardness value of Ni-TiN coatings increased initially and then decreased. The hardness values of Ni-TiN coatings produced at 0.3 T, 0.7 T, and 1.1 T were 704.2 Hv, 817.3 Hv, and 749.6, respectively. This result contributed to an appropriate magnetic intensity, which could reduce the agglomeration of TiN nanoparticles and generate strong dispersion hardening effect, resulting in increased hardness value of coatings [29]. Furthermore, the hardness value of the TiN nanoparticle was larger than that of nickel metal, causing the value of Ni-TiN coatings to increase with high TiN content [30].

### 3.5. XRD Pattern Analysis

**Figure 10:**
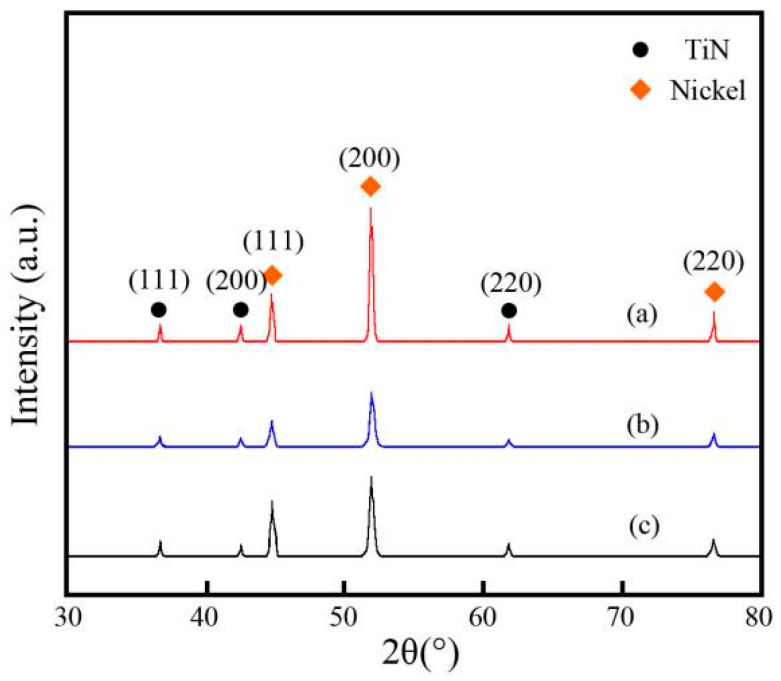
XRD patterns of Ni-TiN coatings produced at various magnetic intensities: (**a**) 0.3 T, (**b**) 0.7 T, and (**c**) 1.1 T.

Figure 10 reveals the XRD pattern of Ni-TiN coatings prepared at different magnetic intensities. The average size of nickel grains in the Ni-TiN coatings are listed in Table 2. It could be seen that the crystal structure of Ni-TiN coatings presented with face-centered cubic (FCC) [31]. The nickel phase and TiN phase both existed in three Ni-TiN coatings. Three diffraction peaks of nickel phase in the Ni-TiN coatings emerged at 44.9°, 52.1°, and 76.8°, corresponding to the crystal planes of (111), (200), and (220), respectively [32]. Meanwhile, the diffraction peaks of the TiN phase in the Ni-TiN coatings appeared at 36.8°, 42.7°, and 61.9°, corresponding to the crystal planes of (111), (200), and (220), respectively (PDF#38-1420) [33]. Furthermore, the diffraction peak intensity of Ni-TiN coatings manufactured at 0.7 T was lower than that of other ones fabricated at 0.3 T and 1.1 T. This result contributed to an appropriate magnetic intensity, which could refine the Ni-TiN coatings surface and further reduce the average size of nickel grain.

According to the calculation of Equation (1), with the growth in magnetic intensity, the average size of nickel grain in the Ni-TiN coatings decreased initially and then increased. When the magnetic intensity rose from 0.3 T to 0.7 T, the average size of the nickel grain reduced from 879.3 nm to 308.4 nm. When the magnetic intensity increased from 0.7 T to 1.1 T, the average size of nickel grain increased from 308.4 nm to 517.6 nm.

### 3.6. Corrosion Resistance Measurement

**Figure 11:**
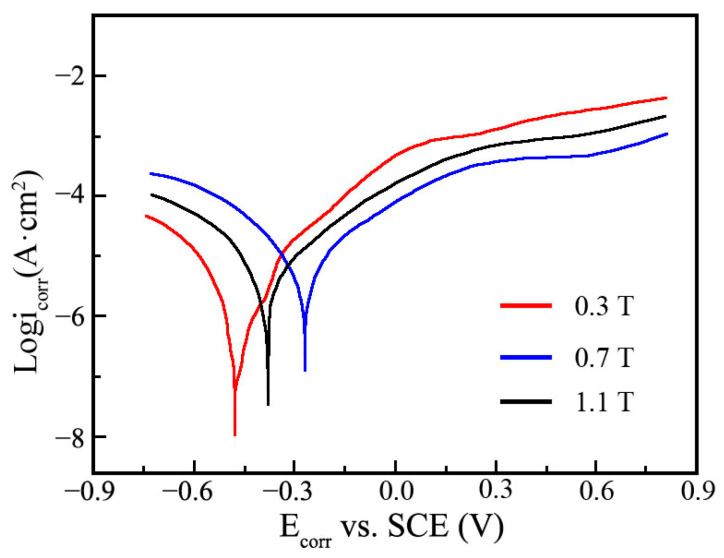
Polarization curves of Ni-TiN coatings fabricated at different magnetic intensities.

Figure 11 indicates the effect of magnetic intensity on the polarization curves of Ni-TiN coatings in 3.5 wt.% NaCl solution. Li et al. [34] and Chen et al. [35] proposed that the nickel matrix coatings had large corrosion current and low corrosion potential, which meant a poor corrosion resistance against 3.5 wt.% NaCl solution. The corrosion current density and corrosion potential of Ni-TiN coatings prepared at 0.3 T were 8.7 μA/cm^2^ and −0.49 V, respectively, demonstrating poor corrosion resistance. By comparison, the corrosion current density and corrosion potential of Ni-TiN coatings fabricated at 0.7 T were 5.4 μA/cm^2^ and −0.26 V, respectively, illustrating the best corrosion resistance. However, when the magnetic intensity was 1.1 T, the corrosion current density of Ni-TiN coatings increased to 7.9 μA/cm^2^, while the corrosion potential decreased to −0.34 V. These findings confirmed that the Ni-TiN coatings prepared at 0.7 T owned excellent corrosion resistance, which was superior to the corrosion resistance of Ni-Al coatings fabricated at electrodeposition [36].

**Figure 12:**
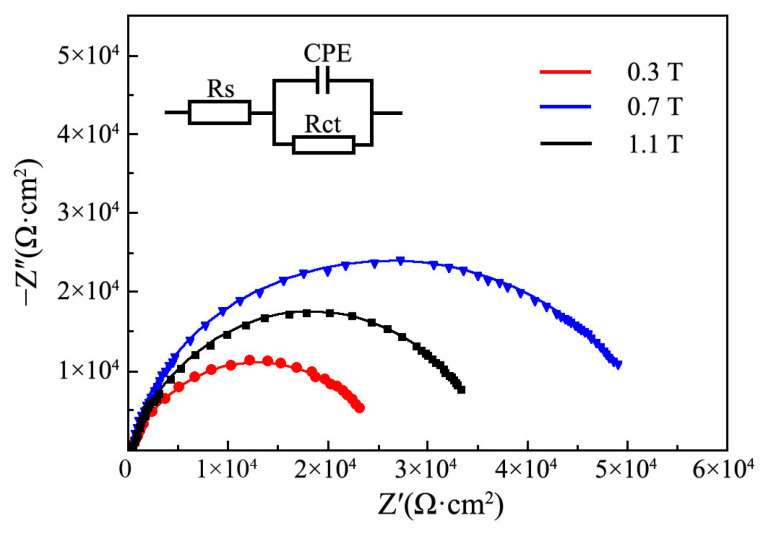
Nyquist plots of Ni-TiN coatings produced at different magnetic intensities.

Figure 12 reveals the Nyquist plots of Ni-TiN coatings fabricated at various magnetic intensities. Hu et al. [37] and Nasri et al. [38] reported that the corrosion resistance of nickel matrix coatings corresponded highly to the impedance value. The Ni-TiN coatings obtained at 0.3 T had the smallest impedance value, corresponding the worst corrosion resistance. By contrast, the impedance value of Ni-TiN coatings manufactured at 0.7 T was the largest, indicating the best corrosion resistance. However, when the magnetic intensity rose to 1.1 T, the impedance value of Ni-TiN coatings decreased, demonstrating that its corrosion resistance was reduced.

**Figure 13:**
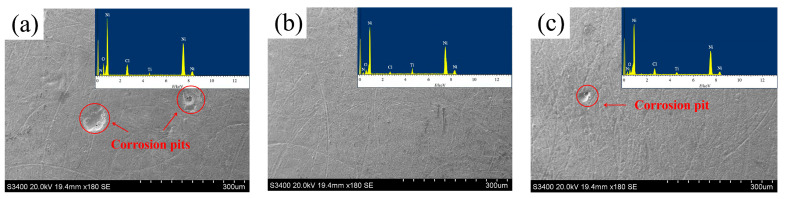
Corrosion surface morphologies and EDS results of Ni-TiN coatings produced at different magnetic intensities: (**a**) 0.3 T, (**b**) 0.7 T, and (**c**) 1.1 T.

Figure 13 presents the corrosion surface morphologies and EDS results of Ni-TiN coatings fabricated at different magnetic intensities. The average corrosive weight losses of Ni-TiN coatings manufactured at various magnetic intensities are listed in Table 3. Zhu et al. [39] and Li et al. [40] found that the corrosion surface morphology of nickel matrix coatings was significantly influenced by the content and distribution of reinforced phase. The obvious corrosion pits were not found on the surface of Ni-TiN coatings prepared at 0.7 T, whereas numerous and large corrosion pits appeared on the surface of Ni-TiN coatings fabricated at 0.3 T. In addition, the average corrosive weight losses of Ni-TiN coatings manufactured at 0.3 T, 0.7 T, and 1.1 T were 12.4 mg, 7.2 mg, and 9.1 mg, respectively.

The reason of this result could be illustrated as follows: (1) The low or high magnetic intensity caused the low TiN content of Ni-TiN coatings, leading to the penetration of NaCl solution for coatings [41]. (2) Meanwhile, the low TiN content of coatings possessed a coarse and uneven surface morphology, resulting in the large contact area between coatings and NaCl solution [42]. (3) Moreover, the appropriate magnetic intensity contributed to the decreased agglomeration of TiN nanoparticles, leading to the uniform distribution of reinforced phase in the coatings, which could obviously prolong the corrosion paths and enhance corrosion resistance [43].

## 4. Conclusions

(1) SEM images presented the numerous and prominent granules, which appeared on the surface of Ni-TiN coatings produced at 0.3 T, while the surface morphology of Ni-TiN coatings manufactured at 0.7 T was dense and smooth. Among three Ni-TiN coatings, the one produced at 0.7 T had the largest cross-sectional thickness at 87.1 μm and the largest adhesion strength at 47.5 N.

(2) EDS results and hardness test revealed that the Ti content and hardness of Ni-TiN coatings manufactured at 0.7 T were the highest at 9.6 wt.% and 817.3 Hv, respectively. Furthermore, XRD patterns showed that the nickel phase diffraction peaks of Ni-TiN coatings fabricated at 0.7 T were broad and wide, demonstrating the fine size of nickel grain.

(3) In comparison to the other two Ni-TiN coatings, the one manufactured at 0.7 T possessed the highest corrosion potential at −0.26 V, the smallest corrosion current at 5.4 μA/cm^2^, and the lowest average corrosive weight loss was 7.2 mg, demonstrating an outstanding corrosion resistance. These conclusions indicated that the optimized magnetic intensity in the electrodeposition process could provide an effective technique to improve the corrosion resistance and expand the Ni-TiN coatings used in the application fields of petroleum and chemical industries.

## Figures and Tables

**Table 1:** Electrolyte component and operation parameter for preparing electrodeposited Ni-TiN coatings.

Composition	Specific
NiSO_4_ (g/L)	190
NiCl_2_ (g/L)	40
H_3_BO_3_ (g/L)	30
TiN concentration (g/L)	6
Cetyltrimethyl ammonium bromide (mg/L)	60
Current density (A/dm^2^)	3
Magnetic intensity (T)	0.3, 0.7 and 1.1
pH	4.8
Electrodeposition time (min)	40
Electrolyte temperature (°C)	45

**Table 2:** Average size of nickel grains in Ni-TiN coatings.

Ni-TiN Specimens	Average Size of Nickel Grains
Obtained at 0.3 T	879.3 nm
Obtained at 0.7 T	308.4 nm
Obtained at 1.1 T	527.6 nm

**Table 3:** Average corrosive weight loss of Ni-TiN coatings manufactured at various magnetic intensities.

Ni-TiN Specimens	Average Corrosive Weight Loss
Obtained at 0.3 T	12.4 mg
Obtained at 0.7 T	7.2 mg
Obtained at 1.1 T	9.1 mg

## Data Availability

The original contributions presented in this study are included in the article. Further inquiries can be directed to the corresponding authors.

## References

[1] Ru J., Jia Y., Jiang Y., Feng J., Zhou R., Hua Y., Wang D. (2017). Modification of ZTA particles with Ni coating by electroless deposition. Surf. Eng..

[2] Li Q.S., Cui J., Yang Y.F., Li J., Zhao Y.H., Yu C.Y., Wang Q.W., Zhang P. (2024). Enhanced surface wettability modification of Al_2_O_3_ for laser cladding ceramic-metal composite coatings. Mater. Today Commun..

[3] Babbar A., Singh G., Singh V., Walia R.S. (2025). Progress and recent developments in carbon nanotube (CNT) based composite coatings for tribological and bio-tribological applications. J. Ind. Eng. Chem..

[4] Gao M.Y., Pei Z.J., Song G.H., Liu Z.Y., Gu W.S., Gong J. (2025). Wear resistance of electro-brush plated Ni/diamond nanocomposite coatings. Diam. Relat. Mater..

[5] Zhao Y.T., Liu S.Q., Guo H., Li H.X., Zhang J.N., Ma Z., Zhong N., Li W.G., Ji V. (2025). Mechanistic insights on the microstructural evolution and tribological performance of the Ni-MoS2 composite coatings electrodeposited under different current densities. J. Mater. Res. Technol..

[6] Razak M.B.A., Liza S., Fukuda K., Tahir N.A.M., Khairunnisa M.P., Yaakob Y. (2025). Investigation of surface morphology, wettability and tribological properties of Ni composite coating added with graphite. Surf. Topogr.-Metrol..

[7] Xu L.T., Li Y.F., Zheng L. (2025). Preparation and mechanical properties of Ni-P-Al2O3-SiO2 composite coatings by pulsed electrodeposition. Bull. Mater. Sci..

[8] Harsha S., Dwivedi D.K., Agarwal A. (2008). Performance of flame sprayed Ni-WC coating under abrasive wear conditions. J. Mater. Eng. Perform..

[9] Zhou C., Li M., Chi J., Wang S.F., Zhang M.Y., Fang M., Ren L.S. (2020). Influence of ball milling process on microstructure and properties of Ni-based coating by laser cladding. Appl. Phys. A-Mater..

[10] Mi P.B., Liu J.L., Zhou Z.P., Zhao H.J., Qi C.J., He J.N. (2023). The dynamic compressive properties of B4C ceramic by plasma spraying multilayer Co/Ni-TiCN coatings. Ceram. Int..

[11] Gyawali G., Dhakal D.R., Joshi B., Choi J.H., Lee S.W. (2019). Evaluation of scratch resistant properties of electroless Ni-P-Al2O3 composite coatings. J. Ceram. Process. Res..

[12] Wen Y.X., Zhao Y., Zhang Z.Y., Wu Y.C., Zhu H., Xu K., Liu Y. (2024). Electrodeposition of Ni-Mo alloys and composite coatings: A review and future directions. J. Manuf. Process..

[13] Xu Y.K., Fan M.Y., Luo Y.Q., Zhao Q.Y., Chen Y.N., Hao J.M. (2021). SiC/TiN Particles Reinforced Ni-Mo Nanocomposite Coating Prepared by Pulse Electrodeposition. Rare Metal Mater. Eng..

[14] Gao M.Y., Liu L.L., Huang Z.X., Jiang C., Wang X., Huang F., Li X.X., Zhang X.H. (2024). Electrodeposition and properties of Ni-W-Ti2AlC composite coatings. Ceram. Int..

[15] Ren A.H., Fu X.Q., Chen X.X., Lin J.R., Cao H.B. (2021). Effect of Current Density on the Properties of Ni-CeO2 Composite Coatings prepared using Magnetic Field-Assisted Jet Electrodeposition. Int. J. Electrochem. Sci..

[16] Liu J.G., Tong S.L., Wang S.H., Wan Z.Y., Xing X., Cui G. (2024). The Preparation and Properties of a Ni-SiO2 Superamphiphobic Coating Obtained by Electrodeposition. Metals.

[17] Guo S.J., Yang Z.G., Deng S.H., Wang S., Wang X. (2022). Effect of Pulse Electrical Parameters on the Microstructure and Performance of Ni-TiN Nanocoatings Prepared by Pulse Electrodeposition Technique. Trans. Indian. Inst. Metals.

[18] Wang J., Cai C., Ma S.L., Cao F.H., Zhang Z., Zhang J.Q. (2010). Preparation and Characterization of Nanostructured Ni-TiN Composite Films. Chin. J. Chem. Phys..

[19] Li X.Y., Zhu Y.Y., Xiao G.R., Feng J., Zhou R., Hua Y., Wang D. (2014). Application of artificial neural networks to predict sliding wear resistance of Ni-TiN nanocomposite coatings deposited by pulse electrodeposition. Ceram. Int..

[20] Azizi-Nour J., Nasirpouri F. (2022). Exploiting magnetic sediment co-electrodeposition mechanism in Ni-Al2O3 nanocomposite coatings. Electroanal. Chem..

[21] Jiang W., Shen L.D., Qiu M.B., Xu M.Y., Tian Z.J. (2018). Microhardness, wear, and corrosion resistance of Ni-SiC composite coating with magnetic-field-assisted jet electrodeposition. Mater. Res. Express.

[22] Reddah I., Ghelani L., Touati S., Lekmine F., Hvizdos P., Devesa S., Boumediri H. (2025). Experimental Investigation and Optimization of the Electrodeposition Parameters of Ni-Al_2_O_3_ Composite Coating Using the Taguchi Method. Coatings.

[23] Zhang W.W., Du S.S., Li B.S., Mei T.Y., Miao Y.C., Chu H.Q., Wang J.J. (2021). Synthesis and characterization of TiN nanoparticle reinforced binary Ni-Co alloy coatings. J. Alloy Compd..

[24] Ji R.J., Han K., Jin H., Li X.P., Liu Y.H., Liu S.G., Dong T.C., Cai B.P., Cheng W.H. (2020). Preparation of Ni-SiC nano-composite coating by rotating magnetic field-assisted electrodeposition. J. Manuf. Process..

[25] Li B.S., Mei T.Y., Chu H.Q., Wang J.J., Du S.S., Miao Y.C., Zhang W.W. (2021). Ultrasonic-assisted electrodeposition of Ni/diamond composite coatings and its structure and electrochemical properties. Ultrason. Sonochem..

[26] Yan H.B., Shen Y.J., Zhao Z.C., Cai R., Chen Q.C., Dong G.F. (2025). Study on Properties of Ni-ZrO_2_ Nanocomposite Coatings Prepared by Pulsed Electrocasting Under the Coupling Effect of Ultrasonic and Magnetic Field. JOM-US.

[27] Dong X.W., Yin B., Zhu C.Y., Wang M., Li W.K., Li Q.D. (2023). Effect of magnetic field intensity on the microstructure and abrasion resistance of magnetic electrodeposited Ni-Co-SiC thin films. Ceram. Int..

[28] Ma C.Y., He H.X., Zhang H.B., Li Z.P., Wei L.X., Xia F.F. (2024). Impact of Magnetic Field Direction on Performance and Structure of Ni-Co-SiC Coatings Fabricated via Magnetic-Field-Induced Electrodeposition. Coatings.

[29] Chaudhari A.K., Singh V.B. (2015). Studies on Electrodeposition, Microstructure and Physical Properties of Ni-Fe/In2O3 Nanocomposite. J. Electrochem. Soc..

[30] Zhang H.B., Li Z.P., Wei L.X., Xia F.F. (2024). The Influence of Magnetic Field Orientation on the Efficacy and Structure of Ni-W-SiC Coatings Produced by Magnetic Field-Assisted Electrodeposition. Coatings.

[31] Xia F.F., Li C.Y., Ma C.Y., Li Q. (2021). Effect of pulse current density on microstructure and wear property of Ni-TiN nanocoatings deposited via pulse electrodeposition. Appl. Surf. Sci..

[32] Li Q., Xia F.F., Liu G.F., Yao L.M. (2022). Microstructure and Properties of Jet Pulse Electrodeposited Ni-TiN Nanocoatings. J. Mater. Eng. Perform..

[33] Xia F.F., Li Q., Ma C.Y., Liu W.Q., Ma Z.P. (2020). Preparation and wear properties of Ni/TiN-SiC nanocoatings obtained by pulse current electrodeposition. Ceram. Int..

[34] Li C.Y., Xia F.F., Yao L.M., Li H.X., Jia X. (2023). Investigation of the mechanical properties and corrosion behaviors of Ni-BN-TiC layers constructed via laser cladding technique. Ceram. Int..

[35] Chen Y.L., Li H.G., Zhu J.C., Fang C., Li Z.Q., Jiang W. (2024). Fabrication of Ni-TiN nanocomposite coatings by ultrasonic assisted jet electrodeposition. Part B J. Eng. Manuf..

[36] Arghavanian R., Bostani B., Parvini-Ahmadi N. (2015). Characterisation of coelectrodeposited Ni-Al composite coating. Surf. Eng..

[37] Hu X.Y., Qu N.S. (2019). Improved Corrosion Resistance of Ni-Co Coatings Prepared by Electrodeposition with Large Centrifugal Acceleration. J. Mater. Eng. Perform..

[38] Nasri K., Ahmed N.A., Issaasi H.K., Hamzaoui A.H., Aliouane N., Djermoune A., Chassigneux C., Eyraud M. (2025). Modification of ZTA particles with Ni coating by electroless deposition. J. Indian Chem. Soc..

[39] Zhu Y.S., Gu C.Q., Wang J.L., Xi X.H., Qin Z.B. (2023). Characterization and corrosion behavior of Ni-Cr coatings by using pulse current electrodeposition. Anti-Corros. Methods Mater..

[40] Li C.Y., Xia F.F., Ma C.Y., Li Q. (2021). Research on the Corrosion Behavior of Ni-SiC Nanocoating Prepared Using a Jet Electrodeposition Technique. J. Mater. Eng. Perform..

[41] Xia F.F., Li Q., Ma C.Y., Gun X. (2020). Preparation and characterization of Ni-AlN nanocoatings deposited by magnetic field assisted electrodeposition technique. Ceram. Int..

[42] Sun J., Du D.X., Lv H.F., Zhou L., Wang Y.G., Qi C.G. (2015). Microstructure and corrosion resistance of pulse electrodeposited Ni-Cr coatings. Surf. Eng..

[43] Zhang R.Y., Yang K., Zhang Y.P., Tao B., Wang S.Q., Cheng Q.L. (2024). Long-term anticorrosion performance of a modifier-free Ni-graphene superhydrophobic coating. Mater. Today Commun..

